# Cryosurgery with Pulsed Electric Fields

**DOI:** 10.1371/journal.pone.0026219

**Published:** 2011-11-07

**Authors:** Charlotte S. Daniels, Boris Rubinsky

**Affiliations:** Department of Mechanical Engineering, University of California, Berkeley, Berkeley, California, United States of America; University of South Florida College of Medicine, United States of America

## Abstract

This study explores the hypothesis that combining the minimally invasive surgical techniques of cryosurgery and pulsed electric fields will eliminate some of the major disadvantages of these techniques while retaining their advantages. Cryosurgery, tissue ablation by freezing, is a well-established minimally invasive surgical technique. One disadvantage of cryosurgery concerns the mechanism of cell death; cells at high subzero temperature on the outer rim of the frozen lesion can survive. Pulsed electric fields (PEF) are another minimally invasive surgical technique in which high strength and very rapid electric pulses are delivered across cells to permeabilize the cell membrane for applications such as gene delivery, electrochemotherapy and irreversible electroporation. The very short time scale of the electric pulses is disadvantageous because it does not facilitate real time control over the procedure. We hypothesize that applying the electric pulses during the cryosurgical procedure in such a way that the electric field vector is parallel to the heat flux vector will have the effect of confining the electric fields to the frozen/cold region of tissue, thereby ablating the cells that survive freezing while facilitating controlled use of the PEF in the cold confined region. A finite element analysis of the electric field and heat conduction equations during simultaneous tissue treatment with cryosurgery and PEF (cryosurgery/PEF) was used to study the effect of tissue freezing on electric fields. The study yielded motivating results. Because of decreased electrical conductivity in the frozen/cooled tissue, it experienced temperature induced magnified electric fields in comparison to PEF delivered to the unfrozen tissue control. This suggests that freezing/cooling confines and magnifies the electric fields to those regions; a targeting capability unattainable in traditional PEF. This analysis shows how temperature induced magnified and focused PEFs could be used to ablate cells in the high subzero freezing region of a cryosurgical lesion.

## Introduction

Minimally and non-invasive surgeries are positioned to transform the field of medicine with shorter hospital stays, reduced surgical trauma, improved immune response and greater precision [1]. These benefits are primarily due to less intrusive procedures and more targeted tissue ablation. Various minimally invasive surgical procedures exist, each with their advantages and disadvantages and particular use. This study explores the feasibility and the advantages of the combined use of two minimally invasive surgical techniques: cryosurgery and pulsed electric fields (PEF).

Cryosurgery is a minimally invasive surgical technique that uses freezing to ablate undesirable tissue [2], [3]. Freezing is induced with cryosurgical probes, cooled by an internally circulated cryogen, inserted into or placed in close vicinity to the undesirable tissue. During cryosurgery the frozen lesion propagates as a function of time outward from the cryoprobe, into the tissue, until the undesirable tissue is completely frozen. The time scale of typical cryosurgical procedures is on the order of magnitude of single to tens of minutes. The temperature distribution in the frozen lesion ranges from the cryosurgical probe temperature at the interface between the cryoprobe and the tissue, to the change of phase temperature for physiological solutions, −0.56°C, on the outer edge of the frozen lesion [4]. Cryosurgery has many advantages. One is that freezing has a profound effect on the physical properties of tissue and therefore cryosurgery can be monitored with virtually every medical imaging modality [2], [5], [6]. Because of cryosurgery's time scale, imaging allows the physician real time control over the extent of freezing with excellent clinical results [7].

Cryosurgery, however, also has limitations. These limitations are related to the mechanism of cell death by freezing and the incomplete cell death in certain ranges of subfreezing temperatures [2], [3], [8], [9], [10]. A recent review [3] points out that: “The tissue temperature is a key factor in causing injury… As a guide for the treatment of neoplasms, the many experiments suggesting that about −20°C is adequate for tissue destruction should be viewed with caution. Certainly extensive tissue damage occurs in the −20 to −30°C range, but tumor cell destruction at the temperature range is uncertain and incomplete.” This means that on the outer rim of the frozen lesion, cells in the temperature range from at least −30°C to −0.56°C can survive the procedure. Medical imaging can only discriminate between frozen and unfrozen tissue. This is problematic because the region seen as frozen via imaging contains a substantial number of surviving cells, especially on the outer rim. Therefore, the accuracy of targeting pathogenic tissue and the reliability of ablation are compromised. Indeed the same review [3] suggests that: “The greatest opportunity for improved efficacy (of cryosurgery) is in the management of the periphery of the cryogenic lesion where cell destruction is partial. Therapy adjunctive to cryosurgery, such as chemical agents, cytotoxic drugs, or irradiation should prove helpful in completing destruction of cells in the periphery of the frozen volume.” Cryoadjunctive strategies to enhance cancer cell sensitivity to freezing is the subject of several investigations [3], [8], [9], [11], [12], [13]. The goal of this study is to explore, with a mathematical model, the feasibility of using pulsed electric fields delivered through the cryosurgical probe during cryosurgery as a cryoadjunctive modality to ablate cells in the temperature range in which they survive freezing.

The PEF modality, also known as electroporation or electropermeabilization, utilizes pulsed electric fields that target the cellular membrane, increasing membrane permeability through the formation of nanoscale defects [14], [15]. The electric field is delivered between two electrodes placed into or in the vicinity of the targeted tissue. Typical PEF parameters currently utilized in medicine and biotechnology employ 0.1 to 10^7^ V/m electric fields, nanosecond to millisecond pulse lengths, and one to several hundred pulses. PEFs can have two different effects on the cell membrane as a function of the electric parameters: reversible and irreversible electroporation. In reversible electroporation the permeabilization of the cell membrane is transient. In tissue, reversible electroporation is used for insertion of DNA molecules into cells for gene therapy [16], [17], [18]. Reversible electroporation is also used with toxic chemicals for tissue ablation in a procedure known as electrochemotherapy [19], [20], [21]. In irreversible electroporation (IRE), the effect of membrane permeabilization is permanent and leads to cell death. Recently, IRE has emerged as an important minimally invasive technique for tissue ablation [22], [23], [24], [25]. An important attribute of the PEF modalities is that their time scale is extremely rapid (nanoseconds to milliseconds). While this is advantageous because it significantly reduces the time of the surgical procedure, it is so rapid that it essentially precludes real time control, which is a major disadvantage. Our hypothesis is that combining PEF with cryosurgery will facilitate real time control over the delivery of the electric fields, for reasons that will be discussed next.

There are several earlier studies that are relevant to the work presented in this paper. We have shown in a previous paper that lowering the temperature of the PEF electrode, and the consequent local cooling of the tissue, has the effect of confining the electric fields to the cooled region [26]. Furthermore, it was found that the cooling magnifies the electric fields in the cooled region relative to electric fields produced by the same pulses in physiological temperature tissue [26]. That paper [26], dealt with temperatures above freezing. To the best of our knowledge, there are currently no studies on the application of pulsed electric fields on frozen biological tissues. However, there are studies on the effects of temperature on electroporation and on the effects of hyperosmolality on electroporation. We believe that these studies should provide us with some guidelines on what to expect during the electroporation of frozen tissue at the outer rim of the frozen lesion during cryosurgery. The biophysical phenomena on which we focus in this paper are related to the process of freezing at the outer edge of the frozen lesion during cryosurgery. It has been established that the process of freezing at the outer edge of the frozen lesion, where cooling rates are low, is characterized by extracellular freezing and shrinking of the cell to accommodate for the difference in osmotic pressure between the interior of the unfrozen cell and the increased osmolality of the exterior frozen milieu [2], [3], [27], [28], [29]. Therefore, during typical cryosurgical protocols, the cells on the outer rim of the frozen region, where cells survive freezing, are: a) unfrozen, b) below freezing temperatures and c) in a hyperosmotic solution.

Several studies have investigated the effects of temperature on PEF. For instance, Diaz investigated the effect of low temperatures on electroporation efficacy. He accomplished electroporation on kidney epithelial cells at temperatures as low as −2°C [30]. Additionally, Gallo demonstrated the trend between temperature and electroporation; as temperature decreases, initial permeabilizing voltage increases [31]. Gallo's study operated on the stratum corneum in the range 0 to 80°C. The same relationship between temperature and electroporation was found in *alga Valonia*, rye leaf protoplast, and mammalian cell lines [32]. From the studies discussed here, we anticipate that: a) electroporation will occur at temperatures that are lower than the lipid phase transition temperature of cells, and b) the strength of the electric field required for electroporation will increase with a decrease in temperature of the electroporated cells. The effect of hyperosmolarity on electroporation was also studied. It was found that when electroporation is carried out in a hypertonic media, cells are permeabilized at a lower voltage than cells maintained in isotonic media and exposed to the same electric pulse parameters [33].

The studies discussed above motivated the foundation of this paper: investigation of a method that combines cryosurgery with PEF to ablate the entire frozen region during cryosurgery, including the tissue in the temperature range of −30°C to −0.56°C, located at the frozen lesion outer rim. Electrical conductivity in ionic solutions is a function of temperature, exhibiting a positive correlation. Consequently, the electrical conductivity of frozen and cooled tissue is substantially lower than those at physiological temperatures. As a result of the temperature dependency of tissue electrical properties we hypothesized that applying PEFs through a cryoprobe during freezing and thawing will concentrate the entire electric field to the low temperature region. Additionally, we hypothesize that cells on the margin of the frozen lesion will experience electroporation. Consequently, the entire frozen lesion will contain cells ablated by the combined mechanisms of cryosurgery and PEF, improving the accuracy of cryosurgical procedures. Since the extent of the frozen region can be monitored in real time by medical imaging and because cryosurgery dominates the time scale of the combination cryosurgery/PEF, the combined treatment, including the effect of the PEF, can be controlled by physicians in real time, through imaging of the frozen lesion.

The goal of this paper is to investigate the combination of cryosurgery and PEF through a mathematical analysis of temperature and electric fields produced by the application of PEFs during cryosurgery. The focus of this study is to examine the effect of changes in temperature and freezing on electric fields and the subsequent implications for affecting cryosurgery treated tissue with PEF.

## Methods

### Theoretical Model

#### Mathematical Formulation

The analysis of the application of PEF during cryosurgery requires the simultaneous solution of the Laplace equation and the Pennes Bioheat equation. The Laplace equation solves for the electrical potential distribution associated with an electric pulse, and the Pennes Bioheat equation solves for the temperature distribution. The form of the Laplace equation utilized in this study is shown below:
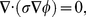
(1)where *σ* is electrical conductivity, and 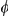 is the electrical potential. The electrical potential is subject to voltage boundary conditions applied at the outer edges of the analyzed domain, and the temperature distribution achieved from the solution of the Bioheat equation.

The temperature distribution was obtained from the solution of a modified Pennes Bioheat equation, which was solved simultaneously as the electrical potential equation. The general bioheat equation has the following form:
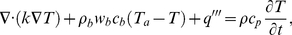
(2)where *k* is thermal conductivity, *T* is temperature, *w_b_* is blood perfusion, *c_b_* is the heat capacity of blood, *T_a_* is arterial temperature, *ρ* is the tissue density, *c_p_* is the tissue heat capacity and 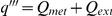. Where *Q_met_* is the metabolic heat generation and 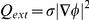
is a term that accounts for Joule heating, where *φ* is electrical potential calculated from Equation 1 and *σ* is the electrical conductivity of the tissue. In this study, it was assumed that there is blood flow and metabolism in the unfrozen tissue while the blood flow and metabolism in the frozen region was set to zero. The effect of Joule heating on the temperature distribution was considered in both the frozen and unfrozen tissues. The equations were solved subject to temperature boundary conditions on the outer surface of the analyzed domain and to the energy balance at the change of phase interface. The initial temperature was assumed to be physiological body temperature.

The mathematical analysis of both the Laplace equation for electrical potential distribution and the various versions of the Pennes Bioheat equation for temperature distribution was performed using a finite element based numerical analysis executed by Comsol Multiphysics (version 4.1). For heat transfer with phase transformation, the enthalpy method was employed to account for the effects of freezing and thawing during cryosurgery. The enthalpy method is a well-established technique for numerical analysis of the phase transition phenomenon in biological media [34]. To validate the use of the commercial software in this work, we tested the convergence of the numerical parameters employed in the analysis against known solutions of the corresponding heat transfer problem. Specifically, a heat transfer with phase change model without electrical parameters was compared to benchmark problems of this kind and validated the approach and results of this paper.

#### Electrical and Thermal Properties

Physiological saline was used as a first order simulation of biological tissue. The electrical conductivity for saline was derived analytically for subzero temperatures using composite theory and the thermodynamic phase diagram for saline. The equation for freezing point depression was used to calculate the volume of solution as a function of temperature. Data from J.J. Arps [33] was curve fitted to calculate the electrical conductivity of the composite medium. The derived electrical conductivity for subzero temperatures was combined with experimental data for higher temperatures [35], resulting in the following piecewise function:
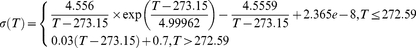
(3)Equation 3 describes the behavior of electrical conductivity in [S/m] as a function of temperature [K], both above and below freezing. The correlation coefficient of this equation relative to experimental data was tabulated to be *r = 0.99989*.

In addition to electrical conductivity, electrical permittivity is also a function of temperature. The following equation [36] was utilized to take into account the temperature dependence of electrical permittivity:
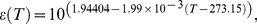
(4)where *ε* is electrical permittivity and *T* is temperature. This equation is valid for low frequency permittivity, experienced by typical PEF pulse parameters, which are in the range of 0.1-20E-3 seconds.

The values for biological tissue utilized in the Pennes Bioheat equation are listed in Table 1 [22] and the thermal properties used are listed in Table 2.

**Table 1:** Temperature dependent properties used in the Pennes bioheat equation for the purpose of solving the temperature distribution due to the application of the cooling probe.

Thermal Conductivity	Blood Perfusion	Blood Heat Capacity	Metabolic Heat	Tissue Density	Tissue Heat Capacity
0.5 [W/mK]	0.5[kg/m^3^s]	3640 [J/kgK]	33800[W/m^3^]	1000 [kg/m^3^]	3750 [J/kgK]

**Table 2:** Temperature dependent properties used in the heat conduction equation for the purpose of solving the temperature distribution due to the application of the cryosurgical probe.

	Density (*ρ*) [kg/m^3^]	Specific Heat (*C_p_*) [J/kg K]	Thermal Conductivity (*k*) [W/m K]	Reference
Frozen	918	2052	2.31	[37]
Unfrozen	997	4179	0.613	[37]

For consistency with the electric field analysis, properties for physiological saline solution were also used to model the thermal behavior of biological tissue. All three values for frozen media in Table 2 were defined at temperatures below freezing, when *T<272.59*. The models defined values for unfrozen media in Table 2 at temperatures above freezing, when *T>273.59*. The transition region between the frozen and unfrozen media was defined using a smoothed Heaviside function. Therefore, the Heaviside function represented a volume fraction of liquid in the frozen media. The term for specific heat was modified to account for latent heat of fusion in order to model the phase transition:
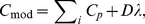
(5)where λ is the latent heat of fusion (333E3 J/kg [40]) and 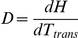. Where *H* is the Heaviside function and *T_trans_* represents the phase transition temperature.

Studies on the effect of temperature on electroporation protocols have revealed a negative correlation between temperature and fields. The electric fields required for producing electroporation increase as temperature decreases. The goal of this study is to investigate the ultimate effects of temperature modulation on cryosurgery/PEF protocols, such as the fields necessary to induce reversible and irreversible electroporation. To accomplish this, data has been extracted from existing literature to produce a correlation between temperature and the fields at transition values between reversible and irreversible electroporation [37], [38]. The equation used in this study was derived from the experimental data acquired in [39] and is given by:
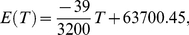
(6)where *T* is temperature [°C] and *E* is electric field [V/m]. This equation was used as an approximate correlation for this study. It was used primarily to demonstrate an accurate methodology, but a substantial amount of additional research is needed to generate precise correlations, which currently have not yet been developed. It should be emphasized that we expect that the hyperosmolarity of the extracellular solution in the high subzero temperature range should reduce the field required for electroporation [32]. Therefore we anticipate that Equation 6 is an upper limit of the electric field required for electroporation at the conditions on the outer rim of the frozen lesion during cryosurgery.

#### Models

**Figure 1:**
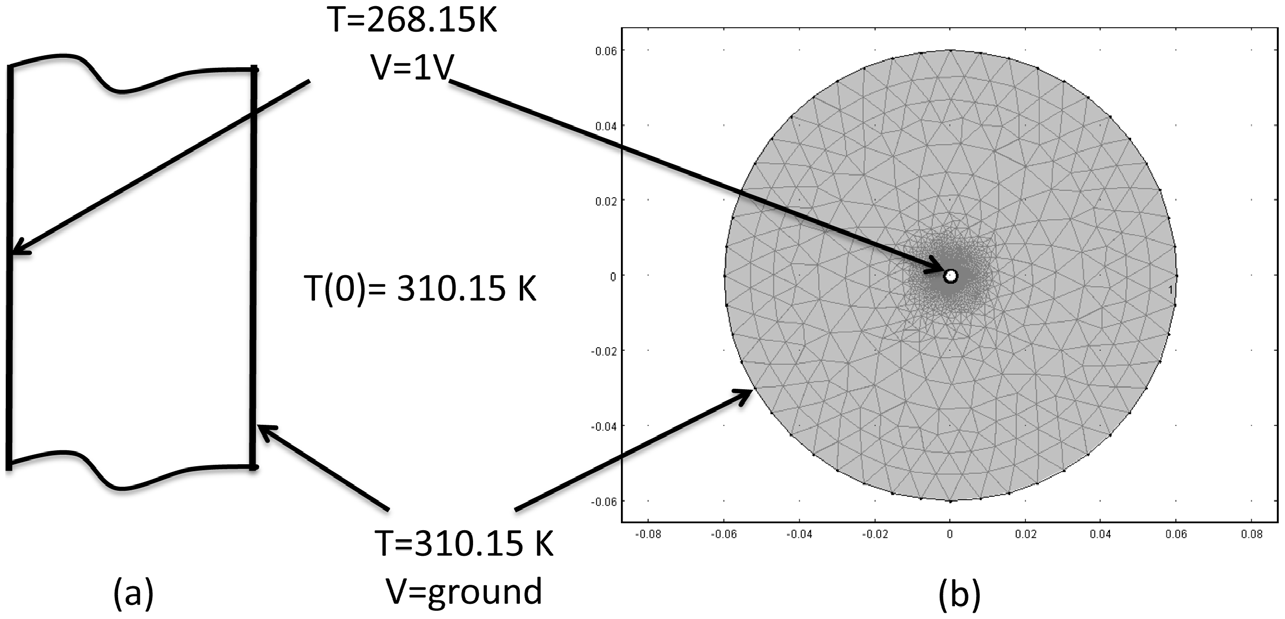
Boundary conditions for the one dimensional Case 1 in a slab-like geometry. Variable resistances *R1* and *R2* represent electric and thermal resistance (in the thermal and electrical equivalent circuits). Boundary conditions utilized in the model are specified at the nodes. The initial temperature was set to physiological temperature, 310.15 K. Figure 1a: geometry of Case 1. Figure 1b: Mesh for Case 2 with one cryoprobe in the center of tissue cylinder. Note that the mesh is extra fine in the vicinity of the probes in order to capture the changes in electric field and temperature that occur due to the effect of the probes.

To extract the most salient biophysical aspects of the analysis, we investigated a simple one-dimensional configuration in Cartesian (Figure 1a) and cylindrical coordinates (Figure 1b). Figure 1a shows a slab like structure of tissue, while Figure 1b shows a cylinder of tissue with a concentric cylindrical cryosurgical probe in the center. The analysis was performed to obtain the temperature distribution and the electric field during freezing and thawing of tissue. The results were compared to a control case in which the electric field was calculated for a constant physiological temperature, 310.15 K. The initial temperature was assumed to be the physiological temperature, 310.15 K. At the onset of freezing, a temperature of 268.15 K was imposed on one surface (the left hand side outer surface in the slab like configuration, Figure 1a, and the central cryoprobe in the cylindrical configuration, Figure 1b). The right hand side of the slab and the outer edge of the cylinder were kept at physiological body temperature, 310.15 K. A temperature of 268.15 K (−5°C) was implemented because in very conservative estimates cell survival occurs at temperatures above 258.15 K in cryosurgery [2], [3], [9], [10]. Therefore, this thermal condition represents the outer margin of a frozen cryosurgical lesion where cells survive freezing [2], [3]; which is a range of temperatures where phenomena related to the Cryo/PEF concept occur. This range enables a test of the conditions in which PEF can ablate cells on the margin of the frozen lesion where frozen cells survive. Furthermore, this is a subzero temperature at which electroporation was observed to occur [30], [39]. The duration of freezing was 90 seconds, after which the cold surface is thermally insulated and natural thawing was induced by constant deep body physiological temperature. The duration of the analyzed thawing period was also 90 seconds. While these periods of time for freezing and thawing are short relative to conventional cryosurgery, they are relevant to the relatively high subfreezing range of temperatures, which is the focus of this analysis. A voltage difference of 1 V between the two outer surfaces of the slab (Figure 1a); and between the cryoprobe surface and the outer surface of the cylindrical tissue (Figure 1b), was used in the calculation of electrical potential to facilitate a general normalized analysis of the electric fields.

An evenly distributed finite element mesh was incorporated into the 6 cm wide slab model. The mesh size was varied in order to validate the accuracy of the solution. The mesh size was refined until the solution was no longer affected by the quality of the mesh. The mesh used in the analysis presented here consisted of roughly 1500 elements.

The second geometry included a typical cryoprobe 3.4 mm in diameter [40], inserted into the center of an infinitely long cylinder of tissue, 6 cm in radius. The finite element mesh utilized for this case incorporated triangular elements, as shown in Figure 1. The element size was smallest adjacent to the cryoprobe, and increased in size as it radiated towards the outer boundary. This was done in order to accurately capture the steep temperature gradient adjacent to the cryoprobe. The mesh was refined until the solution was no longer affected by mesh size. Approximately 3000 elements were utilized to cover a 113 mm^2^ surface area.

## Results and Discussion

### Cartesian Coordinates

**Figure 2:**
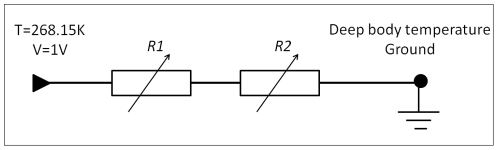
Electric current schematic for the one dimensional 1D Cartesian study in a slab-like geometry. Variable resistances *R_1_* and *R_2_* represent electric and thermal resistance (in the thermal and electrical equivalent circuits). Boundary conditions utilized in the model are specified at the nodes.

The Cartesian coordinate model was chosen for analysis because it provides a simple model which provides fundamental insight into the biophysical processes that occur during the combined application of cryosurgery and PEF. The Cartesian model utilized the boundary conditions specified in Figure 2.

**Figure 3:**
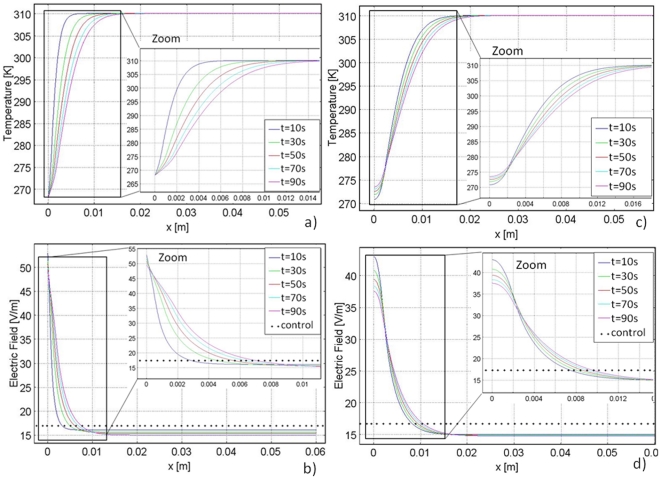
Graphs for the Cartesian coordinate study. Left column: temperature and electric field distribution during 90 seconds of freezing. Right column: temperature and electric field distribution during 90 seconds of thawing. Each line represents a 20 second time increment. The dotted line shows the electric field in tissue held constant at body temperature for the same voltage boundary conditions.

**Figure 4:**
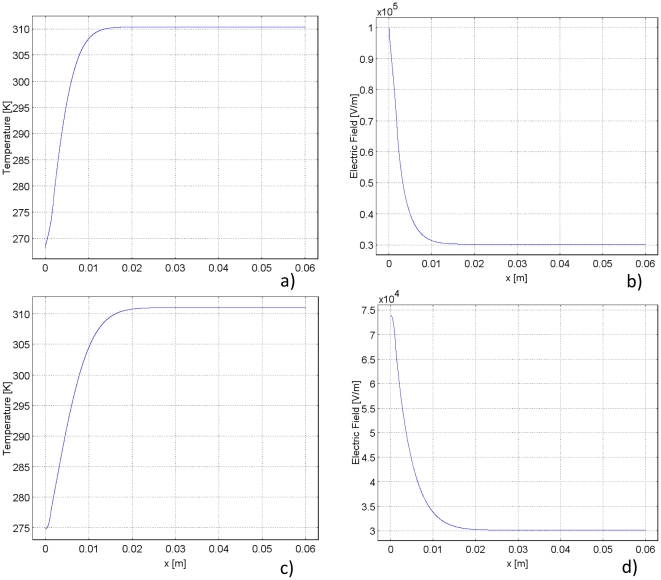
Temperature and electric field plots during freezing and thawing. Left column demonstrates the temperature distribution and right column demonstrates the electric field distribution, both after 90 seconds of freezing with an applied voltage of 2000 V for 90 pulses. The first row illustrates the results due to 90 seconds of freezing and the second row illustrates the results due to 90 seconds of thawing. The trends are similar to those seen in the 1 V cases. However, in the freezing case, the electric field reaches a maximum of 100,000 V/m. In the thawing case the electric field reaches a maximum of 75000 V/m. The temperature rises more quickly at the leftmost plate in both the freezing and thawing cases due to Joule heating.

The temperature distribution during freezing is shown in Figure 3a, and the electric field distribution is shown in Figure 3b. The temperature distribution is as expected, increasing from the low temperature at the cryoprobe surface to body temperature. As time progresses, the low temperatures penetrate further into the tissue due to thermal diffusion. Note that at 272.59 K, the nonlinear behavior indicates the region of phase change. Figures 3a and 3b demonstrate the inversely proportional relationship between temperature and electrical conductivity, as described by Equation 3. Lower temperatures yield a lower ionic conductivity and subfreezing temperatures yield a dramatic decrease in electrical conductivity. Figure 3b illustrates the most important feature of the cryosurgery/PEF combination; because of the increased electrical resistance in the frozen and cooled regions of tissue, the highest electric fields are confined to those regions. Figure 4b also shows that the fields beyond the frozen and cooled regions in the normal tissue are substantially lower than those in the frozen/cooled regions. This suggests that the freezing/cooling has the effect of confining the high electric fields to those regions, as originally predicted.

Figures 2c and 2d illustrate the temperature distribution and the electric field during thawing. Figure 3c shows that adjacent to the cryoprobe, the temperature inches upwards because of the insulating boundary conditions applied by the probe during thawing. If thawing were extended beyond 90 seconds, the temperature graph would continue to equilibrate toward the phase transition temperature. The temperature distribution has a point of inversion, which appears to be stationary in time at about 0.2 cm from the outer surface. This point of inversion corresponds to the position of the change of phase interface.

The temperature history during thawing of frozen media has a peculiarity caused by the effects of the phase transition, which has been studied extensively in the past [41]. This effect appears in Figure 4c. Previous studies have also shown that during thawing of frozen lesions, the temperature of the frozen region increases first to the phase transition temperature, before the frozen tissue begins to thaw, and then remains at this value throughout the thawing process [41]. The explanation for this phenomenon is complex, has been previously discussed in heat transfer papers on cryosurgery, and is not directly relevant to this work. However, the phenomenon is important in the use of cryosurgery with PEF.

Figure 3d shows that the relationship between temperature and electrical conductivity produces an electric field that also has a point of inversion around 0.2 cm, which is the outer edge of the frozen lesion. Figure 3d shows that the electric field in the frozen region drops precipitously during thawing to a constant value of 32 V/m (for the 1 V potential on the cryoprobe). It is apparent that if the temperature in the frozen region during thawing stays at the phase transition temperature, the electrical conductivity in the frozen region will remain constant throughout thawing and (in Cartesian coordinates) the electric field in the frozen region will remain constant as well. This suggests that if PEFs are applied during thawing they will be confined and delivered in the frozen region across cells that are all at the highest possible subfreezing temperature. This could allow for precise design of PEF by utilizing electrical parameters that only affect thawing cells in the frozen tissue at the phase transition temperature.

It is pertinent that the electric field produced during freezing and thawing is compared to a control study. The control study applied the same electrical boundary conditions to tissue held at body temperature. This control study is represented by the dotted horizontal line in Figures 2b (freezing study) and 2d (thawing study). It is evident that the electric field in the frozen/cooled regions is substantially higher than the field produced in the control study in the same region in both freezing and thawing. However, at a distance from the frozen region, in the location of normal body temperatures, the fields are lower than those in the control. This suggests that it should be possible to design cryosurgery/PEF protocols in which the PEF induced cell damage is confined to the frozen/cooled regions and the damage does not extend beyond the cooled regions.

In summary, Figure 3 shows that during both freezing and thawing the electric fields are substantially increased in the frozen/cooled region during cryosurgery with PEF relative to the electric fields in a similar region when PEFs are used with tissue at a constant temperature. The reason for this is illustrated by Figure 2. Figure 2 is an analog representation of the electrical and thermal resistance in the frozen and unfrozen region during cryosurgery. The electrical current flows from one wall of the slab to the other through two resistors in series. The frozen/cooled tissue and the unfrozen tissue equivalent resistances are separated by the phase transition interface. Since the current through the electrodes is constant, it is evident that the resistor of higher magnitude, which in the case in the frozen/cooled region, will experience a higher electric field. The large disparity in electrical conductivity between the frozen/cooled region and the unfrozen tissue confines and magnifies the electric field to the frozen/cooled region. The potential-divider circuit in Figure 2 demonstrates the field enhancement in regions of lower conductivity.

It should be emphasized, that this phenomenon occurs because the electric field vector is normal to the boundary of the two regions of different conductivity. If the electric field were parallel to this boundary, then the equivalent circuit would be two resistors in parallel, rather than in series, and there would be negligible field enhancement or possible field decrease, depending on the relative electrical conductivity of the tissues. The particular configuration developed in this study is a direct consequence of the fact that the cryoprobe serves both as the heat sink and as the electric source. The configurations used in this study were chosen particularly to accomplish this effect.

The plots in Figure 3 were obtained for a normalized voltage of 1 V. Combining Figures 2b and Equation 6 suggests that a voltage of about 2000 V applied to the cryoprobe would be sufficient to ablate the analyzed frozen tissue by PEF. This voltage is typical for what is necessary for tissue ablation with irreversible electroporation. The result of applying 2000 V after 90 seconds of freezing to the model in Figure 3 can be seen in Figure 4a and 4b. As can be seen by this graph, the temperature distribution in the tissue is similar to that in Figure 3, where 1 V was applied. However, one difference is evident; the temperature rises to a higher value over a shorter distance adjacent to the leftmost plate. This is because Joule heating occurs when higher voltages are applied.

The electric field distribution for the application of 2000 V is similar to that of the 1 V case. However, the maximum electric field reached with 2000 V is 100,000 V/m. This results in an electric field distribution in the frozen region above 67,000 V/m, the threshold for irreversible electroporation. Figure 3d and Equation 6 suggest that applying an electric potential above 2000 V is sufficient to cause irreversible electroporation in the thawing region in this model.

Figure 4c and 4d illustrate the temperature and electric field distributions when 2000 V is applied after 90 seconds of thawing. The temperature distribution is similar to that seen in Figure 3c (when 1 V is applied). However, the temperature rises to a higher value over a shorter distance when 2000 V is applied due to Joule heating. The lowest temperature is 275 K in the 2000 V case rather than 274 K as in the 1 V case. The electric field in the 2000 V case has a similar distribution to the 1 V case, except the highest electric field reached in this case is 75,000 V/m. It is important to note that changing the voltage magnitude has a very small effect on the temperature distribution. This is because the energy content in an electroporation pulse is very small relative to the enthalpy of phase transition.

It should be emphasized that in this study the range of subfreezing temperatures used are relatively high, above −5°C. In this temperature range cells survive freezing [2], [3]. Adding PEF during freezing will ablate the cells in the frozen region that survive freezing and thereby making the cryosurgery/PEF treatment produce tissue ablation to the edge of the frozen lesion. This also suggests that the cryosurgery/PEF technique may not require the cryogenic temperatures used in conventional cryosurgery and cryosurgical systems. Therefore, cooling systems using Joule Thomson, solid state thermoelectric systems or conventional refrigeration cycles may be sufficient for tissue ablation with this method. Figure 3 and this analysis corroborate the initial hypothesis of this study: the effect of freezing and low temperatures on electrical conductivity can actually concentrate the electric field to the cooled/frozen region as well as amplify the electric field in that region, which would require substantial lower voltages on the cryosurgery/PEF probes than on conventional PEF probes.

Cryosurgery/PEF protocols can also be designed to induce cell permeabilization via reversible electroporation for the purpose of gene therapy and drug delivery. For example, Figure 3b shows that the field produced by a 1 V potential difference at the cryosurgical probe ranges from about 50 V/m to about 40 V/m between the −5°C and +5°C isotherms. If an attempt were made to combine cryosurgery with electrochemotherapy PEFs, the fields required for reversible electroporation in the liver are about 36,000 V/m. Therefore a pulse of about 700 V will produce reversible electroporation in the region from −5°C to +5°C. However, Figure 3b shows that the same pulse will produce a field of approximately 10,000 V/m in the body temperature region, which should have no effect on the tissue in that location. In contrast, in the control case (at constant body temperature), Figure 3b shows that a pulse of about 1800 V would be needed to produce the fields required for electrochemotherapy and the fields would affect the entire region between the electrodes.

### Cylindrical Coordinates

The purpose of this second study was to examine a more realistic cryosurgical configuration. In Case 2 the geometry consists of a single cryoprobe in a cylindrical section of tissue 6 cm in radius. Geometric details and boundary conditions were previously mentioned in the Models section.

**Figure 5:**
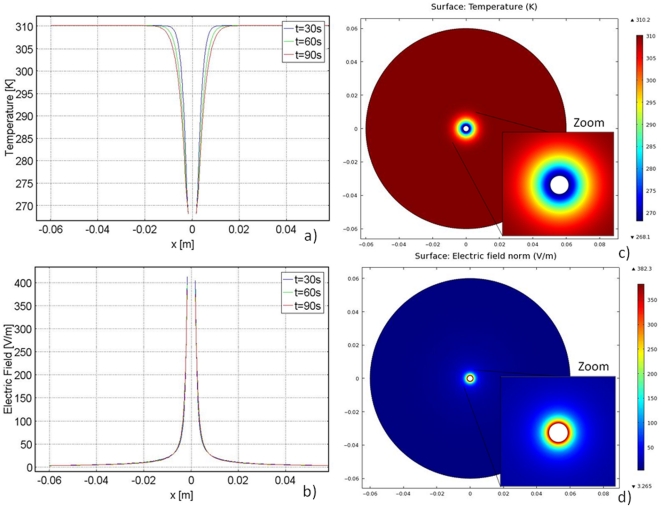
Data for the cylindrical coordinate study. The temperature and electric field distribution are illustrated during 90 seconds of freezing. Left column: graph, right column: surface plot.

Figure 5a illustrates the temperature distribution at a transection along the diameter. Note that in Figure 5a the gap in the plot is the location of the cryoprobe. As in the 1D Cartesian case, due to thermal diffusion, freezing temperatures penetrate further into the tissue with time. And again, nonlinear behavior at 272.59 K indicates the region of phase change.

**Figure 6:**
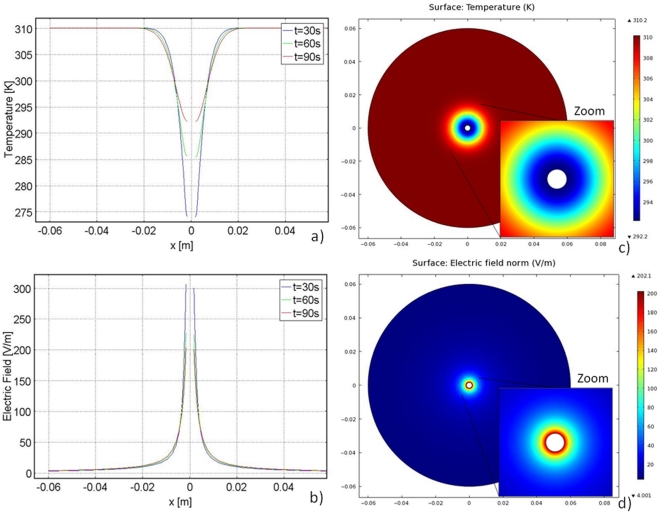
Data for the cylindrical coordinate study. The temperature and electric field distribution are illustrated during 90 seconds of thawing. Left column: graph, right column: surface plot.

The relationship between temperature and electric field is discernable from Figures 4a and 4b. As in the 1D Cartesian study, temperature and electric field are inversely proportional due to the dependence of electrical conductivity on temperature. For this reason, the electric field is highest in the frozen and cooled regions. This result corroborates both the hypothesis of this paper and the results in the 1D Cartesian study. Because of decreased electrical conductivity in the frozen and cooled tissue, the highest electric fields are confined to those regions. Figure 5b shows that the fields beyond the frozen and cooled regions are orders of magnitude lower than those in the frozen/cooled regions. This suggests that freezing/cooling temperatures have the effect of magnifying the high electric fields in those regions. The effect of low temperature on confining the electric field is effectively demonstrated in Figures 6c and 6d. The zoom panel demonstrates that the electric field is confined within the cooled region, which again validates the original hypothesis of this study.

**Figure 7:**
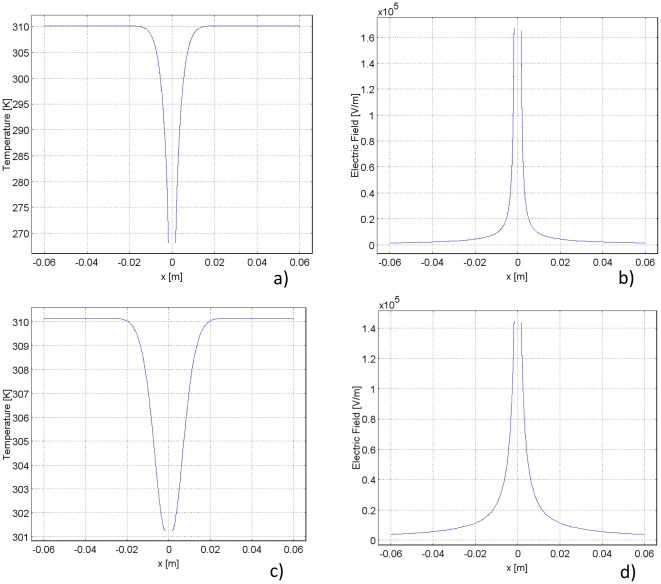
Temperature and electric field distribution during freezing and thawing for the cylindrical coordinate study. Left plot demonstrates the temperature distribution and right plot demonstrates the electric field distribution. The first row illustrates the results after freezing, and the second row illustrates the results after thawing. In each case, a voltage of 400 V is applied for 90 pulses. The trends are similar to those seen in the 1 V cases. However, in the freezing case, the electric field reaches a maximum of 160,000 V/m. In the thawing case, the electric field reaches a maximum of 140,000 V/m. In both instances, the temperature rises higher on the probe due to Joule heating.

Figures 5a and 5b illustrate the temperature distribution and the electric field during thawing. The temperature distribution during thawing with 1D cylindrical symmetry behaves very similarly to the previously discussed temperature distribution in the 1D Cartesian case. Figure 7c shows that temperatures adjacent to the cryoprobe inch upwards as a result of its insulated boundary conditions. The temperature distribution has a stationary point of inversion at 0.6 cm. Figure 6b shows that the electric field also has a point of inversion around 0.6 cm. Figure 6b also demonstrates that the electric field in the frozen/cooled region during thawing drops dramatically, effectively confining the field. Because of the transient nature of the temperature distribution, the electric field also changes with time. For instance, 30 seconds into thawing the highest electric field near the cryoprobe has dropped from 400 V/m at the end of freezing to 300 V/m.

The location of the highest electric field is the same point in space and time as the lowest temperature, which is consistent with Equation 2. Additionally, the electric field decreases in time as thawing progresses and temperatures rise throughout the domain. The slope of the electric field also follows that of the temperature. The slope of the electric field is steepest at the onset of thawing, when temperatures are lowest. But as thawing progresses, and temperatures begin to rise, the slope of the electric field lessens. This indicates the strong relationship between electric field and temperature in the cryosurgery/PEF procedure and the ability of freezing/cold temperatures to confine the electric field. This observation is most evident from Figures 5c and 5d, which depict surface plots of the temperature and electric field distribution, respectively, during the thawing stage of the cryosurgery/PEF procedure. The zoom panels clearly illustrate that the electric field is confined inside the lower temperature regions during thawing.

Figure 5 shows that the electric field in the frozen region is higher than about 150 V/m for a voltage of 1 V on the cryoprobe. Equation 6 suggests that a voltage of about 400 V imposed on the cryosurgical probe is sufficient to ablate the cells with PEF in the analyzed frozen region. A graph of the electric field and temperature distributions with 400 V applied after 90 seconds of freezing can be seen in Figure 7. When 400 V are applied, the temperature rises to a higher value over a shorter distance than when 1 V was applied (Figure 7a) due to the effect of Joule heating. The electric field with 400 V applied demonstrates the same trend as the case with 1 V applied, but reaches a maximum of 160,000 V/m rather than the peak of 400 V/m seen in the 1 V case.

Figure 6 suggests that here also, similar to the Cartesian one dimensional case, the field produced by 1 V potential at the cryosurgery/PEF probe in the thawing frozen lesion will be higher than about 75 V/m. Therefore, Equation 6 suggests that a voltage of about 850 V on the cryosurgery/PEF probe will be sufficient to ablate the frozen cells during thawing with PEF. Figure 7 illustrates the electric field and temperature distributions after 90 seconds of thawing with an applied voltage of 850 V. Due to the higher voltage, Joule heating affects the temperature distribution. In the case of 850 V, the lowest temperature in the domain is 301 K, whereas with an applied voltage of 1 V the lowest temperature is 293 K. The electric field achieved due to 850 V is also much higher, it reaches a peak of 140,000 V/m, whereas the peak in the 1 V case is only 300 V/m.

**Figure 8:**
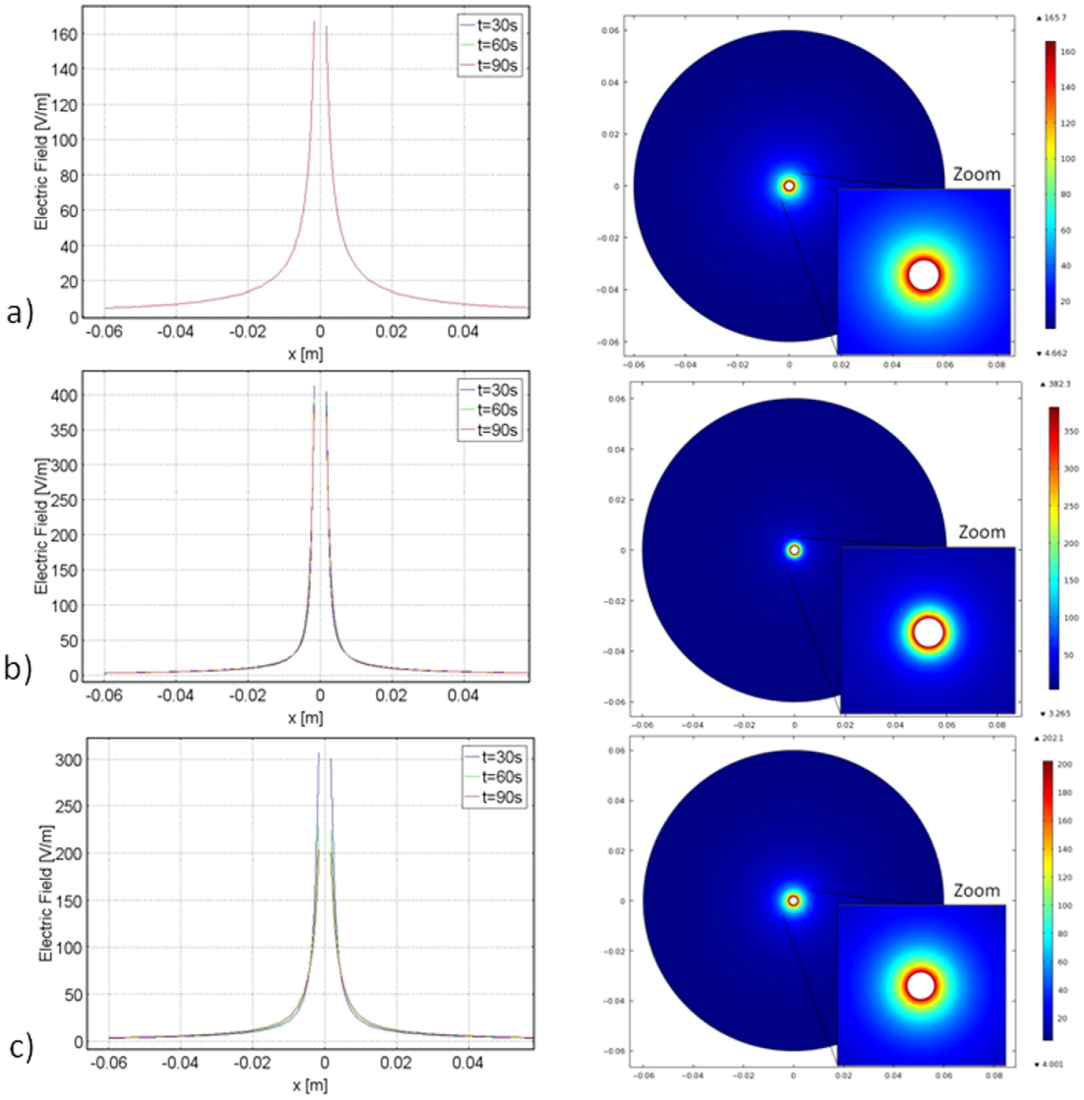
Electric field distribution for the cylindrical coordinate study. From top to bottom row: control, freezing and thawing. Aligned for the purpose of comparison. Right column: surface plot.

The significance of the findings in Figures 4 and 5 is emphasized by a comparison to the control study. The control study applies the same electrical conditions on the cryoprobe as freezing and thawing, but holds the probe and tissue at constant body temperature. The control case represents conventional PEFs delivered by a monopolar probe, which in this case is the cryoprobe. The comparison of the resulting electric fields can be seen in Figure 8. It is clear that the electric field in the frozen/cooled regions during freezing (Figure 8b) and thawing (Figure 8c) are substantially higher than the field produced in the control study in the same region (Figure 8a). The magnitudes of the peak electric field in the freezing and thawing cases are more than double the magnitude of the peak electric field in the control case. However, at a distance from the frozen/cooled tissue, in the region of normal body temperatures, the fields during freezing and thawing are lower than those in the control. This indicates that not only do freezing/cold temperatures amplify the electric field; they also exhibit an effect of targeting the electric field to the cold region. In fact, once the electric field has decayed and reached a constant value, it is zero in the cryosurgery/PEF case, and above zero in the control PEF case.

Figures 7a, 7b and 7c show that the highest electric field occurs, in descending order: freezing, thawing and control. Figures 7a, 7b and 7c also illustrate the ability of cold/freezing temperatures to concentrate the electric field because the most narrowly spread electric field occurs in the freezing case, followed by thawing and then control. This Figure most clearly demonstrates the ability of freezing/cold temperatures to both amplify and direct the electric fields of PEF.

These results suggest that it will be possible to design a cryosurgery/PEF treatment in which the PEF induced cell damage is confined to the frozen/cooled regions and the damage does not extend beyond these regions. The possibility of designing electrical parameters which concentrate the electric field in the frozen/cooled region suggests that utilizing cryosurgical imaging techniques could possibly image cells ablated due to PEFs. For instance, ultrasound is capable of differentiating between frozen and unfrozen media during cryosurgical procedures [2]. Therefore, by concentrating the electric field to the frozen tissue, imaging frozen tissue with ultrasound will also show cells ablated by PEFs in real time during the procedure. This first order theoretical result indicates the potential feasibility of this technology.

Several additional advantages of the cryosurgery/PEF procedure can be theorized as a result of these two studies. Because cells survive these high subzero freezing temperatures, cryosurgery/PEF would retain PEFs' ability to selectively ablate only cellular membranes without affecting the extracellular matrix, when cryosurgery/PEF is used at high subzero temperatures. Another advantage of this combination may be related to elimination of certain side effects and limitations of PEF [41], [42]. The most severe side effects are arrhythmias and involuntary muscle contractions [41], [42] caused by low strength electric fields that expand beyond the PEF treatment zone. The solution to arrhythmias through a synchronizer device is described in [42]. Although strong paralytics such as *cisatracurium* or *rocuronium* [41], [42] and deep anesthesia are used in clinical NTIRE treatments, muscle contractions are still observed in the proximity to the electrodes. Moreover, diaphragm contractions still take place [42]. Our studies show that the combination of cryosurgery with PEF magnify the electric field in the frozen/cooled region and reduces the electric field beyond the frozen/cooled treatment region. Therefore, it could be anticipated that the detrimental effects of PEFs related to weak electric fields that occur beyond the treatment region will be reduced, or perhaps even eliminated.

During the last three decades, cryosurgery has become widely accepted as a minimally invasive surgical technique and is currently used to ablate undesirable tissues in a large number of clinical applications, such as treatment of cancer and arrhythmias. This suggests that the new concept of cryosurgery enhanced by PEF could be more readily accepted among practitioners of cryosurgery. Cryosurgical probes and apparatuses can easily be modified for a cryosurgery/PEF application. The shaft of any cryoprobe is metal and electrically conductive, and therefore could also be used as an electrode. In fact, many cryosurgical probes also have an electrical conduit through the shaft, housing a thermocouple for the purpose of measuring temperatures at the tip of the cryoprobe. This could be one possible path for the electric pulses. Another possible path could be through direct connection to the metal shaft. To deliver the electric pulse only at the thermally active tip of the cryoprobe, it would be sufficient to apply a thin layer of electric insulation along the cryoprobe shaft, as in typical electroporation needles. The pulsed electric field power supply can either be stand alone, or incorporated in the cryosurgery console and connected to the electrically conductive cryoprobe shaft.

There are several ways in which the PEFs could be delivered during cryosurgery. Currently, typical PEF devices operate only between two electrodes at a time, even when multiple electrodes are inserted in the tissue. Therefore, in a first application modality, one thermally active cryoprobe would be connected to one pole of the power supply. The second pole (or ground) could be connected either to a second cryoprobe inserted into the tissue or to a remote ground pad; for instance on the leg, like during radio frequency ablation. The combined Cryosurgery/PEF effect takes place primarily in regions of tissue in which there is freezing and thawing. Therefore, we anticipate that in typical procedures, the PEF will be delivered at the onset of freezing and through freezing and thawing. To take advantage of the ability of medical imaging to detect the extent of freezing, we anticipate that real time imaging with ultrasound or CT will be employed during the Cryosurgery/PEF procedure and in this way ensure that the PEFs are delivered only in the desired cooled area, with real time control over delivery. In this study we have shown only a few of the possible combinations in which this procedure could be used. It is quite obvious that multiple combinations of cryoprobes or different combinations of cold and heat are possible as a way to increase or reduce the magnitude of the PEFs in desired regions of tissue.

### Conclusion

The goal of this study was to evaluate the feasibility and the characteristics of a minimally invasive surgical procedure in which cryosurgery was modulated with PEFs, in the hope that this will improve the effectiveness of cryosurgery and eliminate the problems related to cell survival on the outer rim of frozen tissue as well as produce controlled use of PEF. One dimensional models demonstrated that low and freezing temperatures have a substantial effect on PEF produced electric fields. Furthermore, unanticipated information about the effects of subzero temperature on PEFs was also discovered. This investigation has made clear that both low and subzero temperatures are capable of concentrating the electric field because of the temperature dependence of electrical conductivity. Additionally, this study has demonstrated the ability of low temperatures to increase the magnitude of the electric field.

These results have far reaching implications in terms of imaging strategy and procedural methodology regarding accuracy of treating tissue with cryosurgery. Utilizing cryosurgery/PEF may potentially allow medical imaging of PEF. A further inadvertent consequence of targeted electric fields may be the elimination of muscle contractions during surgery due to the substantial reduction in electric field outside the cryosurgery/PEF treated zone.

Although this is a first order theoretical analysis and additional theoretical and experimental research is needed to further develop this new concept, these primary results act as a promising foundation for future work. For these reasons, this numerical study has indicated the undeveloped potential and the motivation for pursuing cryosurgery/PEF.
